# Longitudinal Analysis of Corneal Biomechanics of Suspect Keratoconus: A Prospective Case-Control Study

**DOI:** 10.3390/bioengineering11050420

**Published:** 2024-04-25

**Authors:** Yan Huo, Xuan Chen, Ruisi Xie, Jing Li, Yan Wang

**Affiliations:** 1 School of Medicine, Nankai University, Tianjin 300071, China; hy13102118953@163.com (Y.H.); chenxuanxuan1122@163.com (X.C.); xieruisi2000@163.com (R.X.); 2 School of Medicine, Northwest University, Xi’an 710199, China; lijing850205@163.com; 3 Clinical College of Ophthalmology, Tianjin Medical University, Tianjin 300070, China; 4 Nankai Eye Institute, Nankai University, Tianjin 300071, China; 5 Tianjin Eye Hospital, Tianjin Key Lab of Ophthalmology and Visual Science, Tianjin Eye Institute, Nankai University Affiliated Eye Hospital, Tianjin 300020, China

**Keywords:** corneal biomechanics, subclinical keratoconus, keratoconus suspects, corneal tomography, refractive surgery

## Abstract

Background: To evaluate the corneal biomechanics of stable keratoconus suspects (Stable-KCS) at 1-year follow-up and compare them with those of subclinical keratoconus (SKC). Methods: This prospective case-control study included the eyes of 144 patients. Biomechanical and tomographic parameters were recorded (Corvis ST and Pentacam). Patients without clinical signs of keratoconus in both eyes but suspicious tomography findings were included in the Stable-KCS group (*n* = 72). Longitudinal follow-up was used to evaluate Stable-KCS changes. Unilateral keratoconus contralateral eyes with suspicious tomography were included in the SKC group (*n* = 72). T-tests and non-parametric tests were used for comparison. Multivariate general linear models were used to adjust for confounding factors for further analysis. Receiver operating characteristic (ROC) curves were used to analyze the distinguishability. Results: The biomechanical and tomographic parameters of Stable-KCS showed no progression during the follow-up time (13.19 ± 2.41 months, *p* > 0.05). Fifteen biomechanical parameters and the Stress–Strain Index (SSI) differed between the two groups (*p* < 0.016). The A1 dArc length showed the strongest distinguishing ability (area under the ROC = 0.888) between Stable-KCS and SKC, with 90.28% sensitivity and 77.78% specificity at the cut-off value of −0.0175. Conclusions: The A1 dArc length could distinguish between Stable-KCS and SKC, indicating the need to focus on changes in the A1 dArc length for keratoconus suspects during the follow-up period. Although both have abnormalities on tomography, the corneal biomechanics and SSI of Stable-KCS were stronger than those of SKC, which may explain the lack of progression of Stable-KCS.

## 1. Introduction

Corneal tomography is the most commonly used screening test before refractive surgery. It can help clinicians make a preliminary diagnosis for first-visit patients [1]. However, many corneas have suspicious tomography abnormalities, which can make it difficult to make a definitive diagnosis and cause considerable confusion for clinicians [2]. Whether the cornea with suspicious tomography abnormalities is a high-risk subclinical keratoconus (SKC) or a stable keratoconus suspect (Stable-KCS) cannot be determined. Longitudinal follow-up is usually required to determine stability. However, during follow-up, the focus is often on corneal tomography information, such as Kmax, corneal thickness, and the corneal posterior surface [3,4,5]. At the same time, there is a risk of vision loss during follow-up. This ‘indefinite’ state is also a major psychological burden for patients [6].

Proteoglycans surrounding collagen fibrils denature in keratoconus and can lead to the fragmentation and degradation of collagen fibrils, thereby altering the local corneal biomechanics and causing morphologic changes. Therefore, corneal biomechanic decompensation may be the reason for morphologic deterioration [7,8,9]. Corneal visualization Scheimpflug technology (Corvis ST; Oculus, Wetzlar, Germany) is presently used to survey and characterize clinical intraocular pressure (IOP) and in vivo corneal biomechanics [10]. The corneal biomechanical parameters provided by Corvis ST, such as SP-A1 (stiffness parameter at first applanation), the DA ratio of 2 mm (corneal deformation ratio within 2 mm), and the integrated radius (calculus of the radius of the reverse concavity), can aid in the early diagnosis of subclinical keratoconus [11,12]. Our previous study found that the biomechanics of SKC are weaker than those of normal corneas [11], which also shows that the weakening of corneal biomechanics is likely to be the reason for morphologic progression. However, for the protection of patients’ vision and quality of life, as well as ethical reasons, any possible progression will receive intervention as soon as possible. Therefore, it is difficult to evaluate the development of the corneal biomechanics of SKC longitudinally. In contrast, it is feasible to conduct longitudinal follow-up observations of Stable-KCS with non-progression. At present, no studies have clarified the biomechanical properties of Stable-KCS. However, analyzing the biomechanical properties of a non-progressing Stable-KCS may provide insight regarding the absence of progression.

Herein, we prospectively analyzed the corneal biomechanics of Stable-KCS without clinical progression and compared them with those of high-risk SKC. To clarify the biomechanical properties of Stable-KCS and screen out sensitive parameters to distinguish Stable-KCS from SKC, we aimed to identify the biomechanical parameters that need to be focused on during the follow-up period to provide clinicians with more biomechanically informed evidence for clinical decision-making.

## 2. Materials and Methods

### 2.1. Ophthalmological Examinations and Data Collection

All patients underwent complete ocular examinations, including non-contact IOP, corrected distance visual acuity (CDVA), corneal tomography, objective and manifest refraction, slit-lamp examination, and biomechanical examination.

Corneal biomechanics were examined using the Corvis ST, which uses an ultra-high-speed Scheimpflug camera to record the corneal deformation. The machine automatically identifies the cornea and captures 140 images in 31 ms after the pulse of the air. During the deformation, the cornea undergoes three states: first flattening, maximum depression, and second flattening. The dynamic corneal response (DCR) parameters obtained from these states are used to characterize corneal biomechanics. The following DCR parameters are used to characterize corneal biomechanics: the weaker the corneal biomechanical properties, the faster its first flattening time and speed after being subjected to an air pulse; the greater the magnitude of the anterior corneal surface deformation, the deeper the indentation, and the smaller the radius of curvature (the deeper the depression, the greater the curvature). The biomechanical parameters used in this study are listed in Supplemental Table S1.

Corneal tomography was performed using Pentacam AXL (Oculus, Wetzlar, Germany). The patient was seated in an upright position in a dark room, positioned with the lower jaw resting on the mandibular rest and the forehead supported by the frontal rest. The examination equipment included a joystick for adjustment and focusing. To capture the necessary pictures, the Scheimpflug camera was utilized, which took 25 tomographic pictures in a 360° rotation in 2 s. These pictures were then reconstructed to generate corneal tomography graphics and obtain corneal morphology data.

The analysis data were derived from “OK” quality original CSV files, and all ophthalmology examinations were performed by experienced doctors.

### 2.2. Participants and Inclusion Criteria

Patients with no clinical signs of keratoconus and meeting any of the following suspicious tomographic features in both eyes were included in the Stable-KCS group: (1) Belin–Ambrósio enhanced ectasia total derivation value (BAD-D): a tomographic index with good clinical applicability; a BAD-D value of >1.6 was considered abnormal [13,14]; (2) abnormal corneal tomography: corneal anterior surface with a bow-tie pattern and skewed radial axes and/or inferior–superior asymmetry (IS-Value) >1.4 diopters [15,16]; and (3) posterior corneal elevation at thinnest point (PTE): a sensitive indicator for the diagnosis of early keratoconus and suggested as a diagnostic point for keratoconus by the global consensus on keratoconus; a PTE of >11 µm was considered abnormal [17,18,19,20,21]. One eye was randomly selected. Longitudinal follow-up of patients in the Stable-KCS group was conducted to define no progression. On an individual level, an experienced clinician (Y.W.) utilized the Pentacam compare function to evaluate the progression. At the statistical analysis level, we performed statistical analyses on tomographic and biomechanical parameters obtained during the initial (first-visit group) and final (last-visit group) visits. The last-visit group was selected to compare the biomechanics with those of the SKC group.

The SKC group included clinical keratoconus contralateral eye, having at least one suspicious tomographic feature without keratoconus clinical signs [15]. The global consensus considers keratoconus to be a bilateral and asymmetric disease [20]. Therefore, even if the tomography of the SKC eye has not changed significantly, its biomechanics have been proven to be relatively weak compared to those of healthy corneas [11,22,23]. This makes it a potential high-risk SKC. All the included patients underwent corneal cross-linking (CXL). The latest preoperative data were selected for case-control analysis.

All included patients had no keratoconus clinical signs (Vogt’s striae, epithelial or subepithelial scarring, Fleischer’s ring, and corneal stromal thinning), clear corneas, and a CDVA of ≥20/20. They were asked to stop wearing contact lenses at least 2 weeks before the evaluation. Patients with a history of eye-associated pathology, injury, or operation were excluded.

### 2.3. Statistical Analysis

The Kolmogorov–Smirnov test was used to evaluate data normality. Continuous variables are expressed as the mean ± standard deviation. An independent sample t-test compared the first- and last-visit corneal parameter differences in the Stable-KCS group. The Kruskal–Wallis test was performed to compare the baseline information and corneal biomechanical parameters between the Stable-KCS and SKC groups; after the Bonferroni correction, a *p*-value < 0.016 indicates a significant difference. Intergroup differences were compared after using a multivariate generalized linear model to correct for central corneal thickness (CCT), a potentially confounding factor. The corneal biomechanical parameter distribution trend among the groups was visualized using a scatter diagram. Receiver operating characteristic (ROC) curves were used to analyze the ability of DCR parameters to differentiate Stable-KCS from SKC. Calculated each parameter’s sensitivity, specificity, and cut-off values, with the area under the ROC curve approaching one, indicating excellent ability. All the statistical analyses were performed using the statistical software SPSS 26.0 (IBM Corp., Armonk, NY, USA). Statistical significance was set at *p* < 0.05. 

## 3. Results

In total, 144 eyes from 144 patients were included in this study, and baseline data are shown in Table 1. The IOP and age showed no significant differences between the Stable-KCS and SKC. There were no significant differences in the PTE, IS-Value, or BAD-D between the two groups; however, the SKC group Stress–Strain Index (SSI) was lower than Stable-KCS (*p* = 0.012).

### 3.1. Suspicious Cornea Analysis

The Stable-KCS group underwent a mean follow-up of 13.19 ± 2.41 months, and none of the tomographic and biomechanical parameters were statistically different between the first and last follow-ups (Table 1 and Table 2).

### 3.2. Corneal Biomechanics Analysis

Sixteen biomechanic parameters were selected based on an earlier study [11]. The DCR parameter differences (Table 3) between the Stable-KCS and SKC groups were analyzed. Stable-KCS had 14 DCR parameter differences with the SKC groups (*p* < 0.016). After CCT correction, significant differences were observed in 15 DCR parameters (*p* < 0.05).

**Figure 1:**
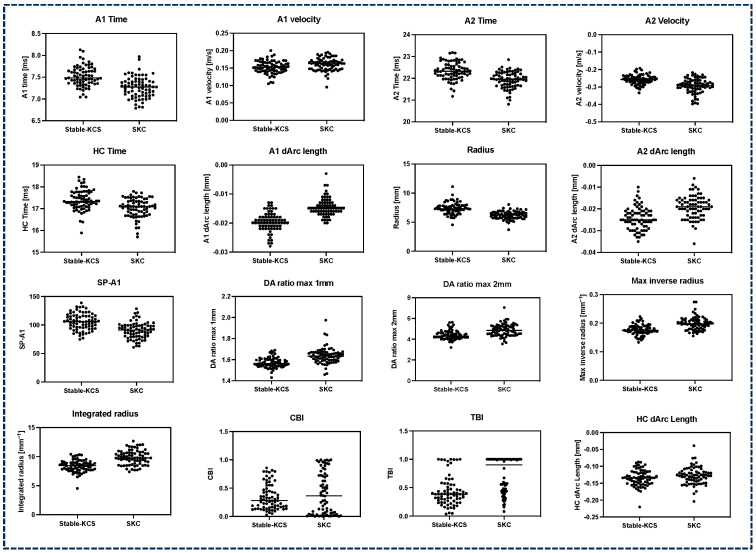
Scatter diagram of corneal biomechanical parameters between the Stable-KCS and SKC groups. Stable-KCS, stable keratoconus suspects (*n* = 72); SKC, subclinical keratoconus (*n* = 72).

Figure 1 shows the corneal biomechanical parameter distribution in the Stable-KCS and SKC groups.

### 3.3. Receiver Operating Characteristic Curve Analysis

**Figure 2:**
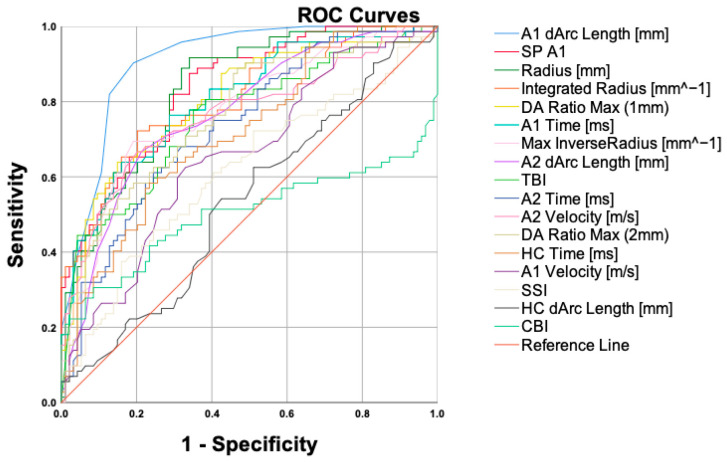
ROC curves for the corneal biomechanical parameters.

The distinguishing abilities of the DCR parameters are shown in Figure 2. The A1 dArc length (Table 4) had the strongest distinguishability (area under the ROC [AUROC] = 0.888) between Stable-KCS and SKC, at a cut-off value of −0.0175 reaching 90.28% sensitivity and 77.78% specificity. 

## 4. Discussion

This study prospectively analyzed corneas with mild tomographic abnormalities to evaluate the biomechanical properties and SSI of these corneas after no progression. We discovered that, despite the presence of tomographic abnormalities, the biomechanical and material properties of Stable-KCS were stronger than those of SKC. This finding may provide an explanation for the non-progression observed in Stable-KCS. Meanwhile, we found the A1 dArc length had the best ability to distinguish Stable-KCS from SKC at a cut-off value of −0.0175 (AUROC = 0.888), with a sensitivity and specificity of 90.28% and 77.78%, respectively. This provides a crucial reference for clinicians to evaluate the risk of progression of the cornea and possible CXL management during the follow-up period.

Currently, corneal tomography is widely used in clinical practice for the early diagnosis of high-risk keratoconus. Although corneal tomography has shown good diagnostic performance [24,25], patients may have congenital, stable corneal morphology abnormalities rather than disease states. Therefore, diversified corneal biomechanical evaluations are necessary for the early and accurate diagnosis of such suspected patients. Another FDA-approved biomechanical characterization device used in clinical practice is the Ocular Response Analyzer, which can provide information on corneal hysteresis (CH), representing the biomechanical properties of the cornea [26]. However, due to the strong relationship between CH and corneal thickness, it may be affected by corneal thickness and cannot accurately represent the stiffness properties of the cornea. CH was considered only to reflect the hysteresis of the cornea [27]. Therefore, this study utilized another in vivo corneal biomechanical testing instrument, the Corvis ST.

In this study, the Stable-KCS group was followed up for 13.19 ± 2.41 months. Their tomographic parameters, such as IS-Value, PTE, BAD-D, Kmax, and corneal thickness, did not change during the follow-up period. The analysis of 16 biomechanical parameters showed no significant changes (*p* > 0.05), indicating the stability of Stable-KCS during the follow-up period. Previous studies have suggested that weaker biomechanical properties are a potential factor contributing to early keratoconus progression [12,23,28]. However, owing to the need to protect patient vision, it is difficult to conduct longitudinal follow-ups on high-risk SKC to further clarify whether the weakening of biomechanical properties is the cause of disease progression. Therefore, to protect vision, we compared the biomechanical properties of Stable-KCS with those of SKC to clarify the role of biomechanics in maintaining stability.

We compared 16 biomechanical parameters between the Stable-KCS and SKC groups and found a significant difference in 14 of these DCR parameters (*p* < 0.016). As corneal biomechanics were affected by corneal thickness [29], the CCT differed between the two groups. We further adjusted the CCT for analysis and found that 15 corneal biomechanical parameters were significantly different between the two groups (*p* < 0.05). Both groups had suspicious tomographic abnormalities; however, the biomechanical properties of Stable-KCS were still stronger than those of SKC, which suggests that stronger corneal biomechanics may explain the lack of progression.

Simultaneously, we evaluated the SSI, which reflects the corneal material properties, between the two groups. SSI is a material stiffness parameter calculated by finite element models [30]. It is used to evaluate the in vivo stress-strain behavior of the cornea [31]. Studies have shown that the SSI is not affected by confounding factors, such as corneal thickness and IOP, and is only correlated with age [30]. There was no statistical difference between the two groups; however, the SKC group SSI was lower than that in the Stable-KCS group (*p* = 0.012). This result indicates that even if there are tomographic abnormalities in both groups, the material properties of SKC have changed. This confirms that the material properties (such as collagen fibers) in early keratoconus may first be altered, further leading to morphologic progression [28].

In our study, the A1 dArc length showed the strongest distinguishing ability (AUROC = 0.888), which had a sensitivity and specificity of 90.28% and 77.78%, respectively, at the cut-off value of −0.0175. The A1 dArc length reflects the alteration from the initial state to the first flattening in the anterior surface. The greater the A1 dArc length, the weaker the corneal biomechanics. SP-A1 demonstrated the second-best distinguishing ability (AUROC = 0.848). SP-A1 is a stiffness index introduced by Roberts et al. [32]. Studies have shown that SP-A1 can characterize the cornea biomechanics in vivo and provide an auxiliary diagnosis for early keratoconus [12]. Our results indicate that, even in the presence of tomographic abnormalities, relying on corneal biomechanical parameters can help distinguish high-risk and stable corneas. Therefore, it is crucial to focus on these two biomechanical parameters when monitoring keratoconus suspects; if the cut-off value is exceeded, keratoconus suspects are likely to progress, which provides novel indicators for clinicians for early intervention and subsequent monitoring in patients suspected of keratoconus during their first visit.

It is worth noting that the current clinical diagnostic parameters, the tomographic biomechanical index (TBI) (AUROC = 0.761) and the Corvis biomechanical index (CBI) (AUROC = 0.502), do not show good distinguishing ability. We consider CBI a machine learning-generated parameter to detect the clinical keratoconus in normal corneas [14]. The data used to train the CBI model primarily come from keratoconus exhibiting significant clinical signs and topography changes. As a result, it may have limited efficacy for accurately distinguishing patients with suspicious abnormal tomography findings but without clinical signs of keratoconus. The TBI, which incorporates the CBI and BAD-D, was introduced as a diagnostic tool for early keratoconus using machine learning algorithms [33]. The original study determined a cut-off value of 0.29, which demonstrated good performance in distinguishing early keratoconus. However, in this study, the BAD-D values were higher in both groups, resulting in the limited ability of TBI to differentiate between the two groups. Machine-learning-based algorithms should be utilized within their intended scope [34]. While they can assist clinicians in detecting diseases more effectively and at an earlier stage, it is crucial to employ them in appropriate clinical scenarios. Therefore, a straightforward evaluation of biomechanical parameters that directly reflect corneal biomechanical properties can demonstrate good discriminative ability. 

In the future, in addition to using corneal tomography for the early screening of patients suspected of high-risk keratoconus, researchers should evaluate the corneal biomechanical properties. Further, additional prospective cohort studies on such abnormal corneal tomography findings are required to determine whether patients with weak corneal biomechanical properties have experienced definite disease progression. Furthermore, corneal biomechanical parameters and artificial intelligence can be combined to introduce more sensitive diagnostic indicators. When patients with suspected abnormal tomography findings and weak biomechanical properties are first evaluated, clinical characteristics, such as age, eye habits, and astigmatism, should be considered to make a preliminary judgment of whether CXL is needed. For patients who have not undergone CXL but have weak corneal biomechanical properties, follow-up evaluation should be conducted within 3–6 months to identify possible disease progression as early as possible, implement early intervention, and protect patient vision.

Our study had some limitations. Owing to the lack of a uniform clinical standard for the follow-up time of keratoconus, we only followed up patients with Stable-KCS for one year to determine whether there were any alterations [3,5]. In the future, we will perform further follow-up on these patients for a longer period to determine their stability. SKC is considered a high-risk progressive condition [35,36], and for early prevention of progression and to protect patient vision, SKC undergoes CXL after short-term follow-up. Therefore, we could not perform long-term follow-ups on SKC to clarify the subsequent changes in their biomechanical properties. Finally, the corneal biomechanical properties measured by Corvis ST are influenced by factors such as IOP, corneal thickness, age, sex, race, and continuous measurement [10,29,37,38]. This study controlled for confounding factors, such as IOP, corneal thickness, and age; we will attempt to incorporate patients of different races for verification in the future.

## 5. Conclusions

The sensitivity and specificity of the A1 dArc length in distinguishing Stable-KCS from SKC reached 90.28% and 77.78%, respectively. Therefore, the A1 dArc length can assist clinicians in making preliminary clinical decisions on first-visit keratoconus suspects. It can also be used as one of the important biomechanical parameters for the clinical monitoring of progression to promptly detect potential changes. Meanwhile, we found that the biomechanical properties and SSI of Stable-KCS were stronger than those of SKC. This suggests that better biomechanical and material properties may be one of the reasons for its non-progression. However, this requires verification and further histopathological studies.

## Figures and Tables

**Table 1:** Baseline information of the participants.

	First (*n* = 72)	Last (*n* = 72)	SKC (*n* = 72)	*p*-Value
Age (y)	23.58 ± 5.24	24.53 ± 5.32	23.19 ± 5.52	*p* = 0.209
Sex (F/M)	25/47	25/47	28/44	-
BCVA	≥20/20	≥20/20	≥20/20	-
IOP (mmHg)	16.34 ± 1.96	16.36 ± 1.90	16.13 ± 1.39	*p* = 0.562
CCT	537.78 ± 24.45	538.64 ± 24.01	508.65 ± 29.78	*p* ^a^ = 1.000 *p* ^b^ < 0.001 * p ^c^ < 0.001 *
TCT	532.01 ± 24.37	532.75 ± 24.65	500.28 ± 29.58	*p* ^a^ = 1.000 *p* ^b^ < 0.001 * p ^c^ < 0.001 *
Kmax	45.51 ± 1.72	45.58 ± 1.78	44.91 ± 1.18	*p* ^a^ = 1.000 *p* ^b^ = 0.045 *p* ^c^ = 0.032
BAD-D	2.24 ± 0.39	2.34 ± 0.41	2.50 ± 0.76	*p* = 0.122
PTE	10.94 ± 3.56	11.21 ± 4.29	12.71 ± 5.51	*p* = 0.129
IS-Value	1.49 ± 0.51	1.52 ± 0.43	1.67 ± 0.58	*p* = 0.438
SSI	0.89 ± 0.15	0.89 ± 0.15	0.83 ± 0.14	*p* ^a^ = 1.000 *p* ^b^ = 0.008 * p ^c^ = 0.012 *

First, first-visit stable keratoconus suspects; Last, last-visit stable keratoconus suspects; SKC, subclinical keratoconus; F, female; M, male; BCVA, best-corrected visual acuity; BAD-D, Belin–Ambrósio enhanced ectasia display total deviation value (D); CCT, central corneal thickness; IOP, intraocular pressure; Kmax, maximum keratometry; PTE, posterior elevation at the thinnest point; IS-Value, 3 mm inferior-superior mean asymmetry difference; TCT, thinnest corneal thickness; SSI, Stress–Strain Index. * *p* values for comparison of the SKC, first, and last groups (*p*-values < 0.016 indicate a statistically significant difference). ^a^ Compared between the first and last groups. ^b^ Compared between the First and SKC groups. ^c^ Compared between the Last and SKC groups.

**Table 2:** Corneal biomechanical parameters between the first and last visits in the stable keratoconus suspects group.

Parameters	First-Visit (*n* = 72)	Last-Visit (*n* = 72)	*p*-Value
A1 time (ms)	7.48 ± 0.28	7.54 ± 0.30	0.209
A2 time (ms)	22.31 ± 0.35	22.34 ± 0.40	0.710
HC time (ms)	17.47 ± 0.52	17.39 ± 0.46	0.316
A1 velocity (m/s)	0.15 ± 0.02	0.15 ± 0.02	0.753
A2 velocity (m/s)	−0.26 ± 0.03	−0.26 ± 0.03	0.768
A1 dArc length (mm)	−0.019 ± 0.003	−0.020 ± 0.003	0.579
A2 dArc length (mm)	−0.025 ± 0.007	−0.024 ± 0.005	0.662
HC dArc length (mm)	−0.135 ± 0.019	−0.134 ± 0.024	0.895
Radius (mm)	7.33 ± 0.72	7.37 ± 0.99	0.770
Integrated radius (mm^−1^)	8.45 ± 0.91	8.40 ± 1.02	0.737
Max inverse radius (mm^−1^)	0.17 ± 0.02	0.18 ± 0.02	0.469
DA ratio max (1 mm)	1.57 ± 0.04	1.57 ± 0.05	0.580
DA ratio max (2 mm)	4.37 ± 0.39	4.40 ± 0.44	0.696
SP-A1	105.63 ± 15.28	105.86 ± 14.44	0.929
CBI	0.35 ± 0.20	0.34 ± 0.23	0.806
TBI	0.49 ± 0.28	0.44 ± 0.26	0.296

*p*-values are for the comparison of the first and last visit stable keratoconus suspects group (*p* < 0.05 indicates a statistically significant difference).

**Table 3:** Comparison of corneal biomechanics between stable keratoconus suspects and subclinical keratoconus.

Parameters	Stable-KCS	SKC	* *p*-value (Stable-KCS vs. SKC)	^a^ *p*-value (Stable-KCS vs. SKC)
A1 time (ms)	7.54 ± 0.30	7.26 ± 0.24	<0.001 *	<0.001 *
A2 time (ms)	22.34 ± 0.40	22.19 ± 0.38	<0.001 *	<0.001 *
HC time (ms)	17.39 ± 0.46	17.03 ± 0.44	<0.001 *	<0.001 *
A1 velocity (m/s)	0.15 ± 0.02	0.16 ± 0.02	0.007 *	<0.001 *
A2 velocity (m/s)	−0.26 ± 0.03	−0.29 ± 0.04	<0.001 *	<0.001 *
DA ratio max (1 mm)	1.57 ± 0.05	1.64 ± 0.08	<0.001 *	<0.001 *
DA ratio max (2 mm)	4.40 ± 0.44	4.87 ± 0.59	<0.001 *	<0.001 *
Radius (mm)	7.37 ± 0.99	6.19 ± 0.69	<0.001 *	<0.001 *
HC dArc length (mm)	−0.134 ± 0.024	−0.122 ± 0.058	0.098	<0.001 *
A1 dArc length (mm)	−0.020 ± 0.003	−0.014 ± 0.003	<0.001 *	<0.001 *
A2 dArc length (mm)	−0.024 ± 0.005	−0.019 ± 0.005	<0.001 *	<0.001 *
Integrated radius (mm^−1^)	8.40 ± 1.02	9.86 ± 1.23	<0.001 *	<0.001 *
Max inverse radius (mm^−1^)	0.18 ± 0.02	0.21 ± 0.07	<0.001 *	<0.001 *
SP-A1	105.86 ± 14.44	89.68 ± 12.76	<0.001 *	<0.001 *
CBI	0.34 ± 0.23	0.41 ± 0.36	0.978	0.077
TBI	0.44 ± 0.26	0.71 ± 0.31	<0.001 *	<0.001 *

SKC, subclinical keratoconus; Stable-KCS, stable keratoconus suspects; * *p*-values for comparison between the Stable-KCS and SKC groups (*p*-values < 0.05 were considered statistically significant). ^a^ *p*-values after central corneal thickness adjustment (*p*-values < 0.05 indicate a statistically significant difference).

**Table 4:** Diagnostic accuracy of biomechanical parameters in stable keratoconus suspects and subclinical keratoconus.

	Cutoff	AUROC	95% CI	Sensitivity	Specificity
A1 dArc length [mm]	−0.0175	0.888	0.834–0.942	90.28%	77.78%
SP-A1	102.56	0.848	0.786–0.911	86.11%	72.22%
Radius [mm]	6.9945	0.847	0.783–0.910	91.67%	73.61%
Integrated radius [mm^−1^]	9.2175	0.811	0.742–0.880	72.22%	81.94%
DA ratio max (1 mm)	1.6131	0.800	0.727–0.873	63.89%	84.72%
A1 time [ms]	7.319	0.786	0.713–0.860	61.11%	84.72%
Max inverse radius [mm^−1^]	0.1905	0.782	0.706–0.857	69.44%	81.94%
A2 dArc length [mm]	−0.0205	0.776	0.700–0.852	66.67%	80.56%
TBI	0.427	0.761	0.683–0.839	77.78%	63.89%
A2 time [ms]	22.117	0.760	0.683–0.838	68.06%	73.61%
A2 velocity [m/s]	−0.2765	0.749	0.669–0.829	68.06%	75.00%
DA ratio max (2 mm)	4.312	0.752	0.672–0.832	88.89%	51.39%
HC time [ms]	17.117	0.701	0.617–0.786	58.33%	73.61%
A1 velocity [m/s]	0.1575	0.635	0.545–0.726	62.50%	65.28%
SSI	0.7625	0.620	0.527–0.712	37.50%	86.11%
HC dArc length [mm]	−0.1325	0.571	0.477–0.665	62.50%	55.56%
CBI	0.826	0.502	0.401–0.603	20.83%	98.61%

AUROC, area under the receiver operating characteristic curve; CI, confidence interval.

## Data Availability

The data presented in this study are available from the corresponding author upon reasonable request.

## Supplementary Materials

The following supporting information can be downloaded at: https://www.mdpi.com/article/10.3390/bioengineering11050420/s1. Supplemental Table S1: Corneal biomechanical parameters provided by the Corvis ST.
