# Supplementary material for: Longitudinal Analysis of Corneal Biomechanics of Suspect Keratoconus: A Prospective Case-Control Study
Source: Bioengineering (Basel). 2024 Apr 25;11(5):420. doi: 10.3390/bioengineering11050420 (PMC11118031; doi:10.3390/bioengineering11050420)
Supplement: Supplementary file 1 [file bioengineering-11-00420-s001.zip › bioengineering-2928054-supplementary.pdf]

Supplemental Table S1. Corneal biomechanical parameters provided by the Corvis ST

**Parameters**	**Definitions**
A1 and A2 Time	Time to reach the first and second applanation
HC Time	Time to reach the highest concavity
A1 and A2 Velocity	Velocity at the first and second applanation
DA Ratio Max (1 mm/2 mm)	Corneal deformation ratio between corneal apex and corneal apex within 1 mm/2 mm
Radius	Radius of curvature at corneal apex at maximum concavity
Max Inverse Radius	Maximum value of the radius of the reverse concave surface
Integrated radius	Calculus of the radius of the reverse concavity
A1 and A2 dArc Length	Arc length change from the initial state to the first and second applanation
HC dArc Length	Arc length change from the initial state to the highest concavity
SP-A1	Stiffness parameter at first applanation
CBI	Corvis biomechanical index
TBI	Tomographic biomechanical index
SSI	Stress-Strain Index
